# Functional composition of ant assemblages in habitat islands is driven by habitat factors and landscape composition

**DOI:** 10.1038/s41598-021-00385-5

**Published:** 2021-10-25

**Authors:** Balázs Deák, Ferenc Báthori, Gábor Lőrinczi, Zsolt Végvári, Dávid D. Nagy, Szabolcs Mizser, Attila Torma, Orsolya Valkó, Béla Tóthmérész

**Affiliations:** 1 Lendület Seed Ecology Research Group, Institute of Ecology and Botany, Centre for Ecological Research, Alkotmány út 2-4, Vácrátót, 2163 Hungary; 2 Lendület Landscape and Conservation Ecology, Institute of Ecology and Botany, Center for Ecological Research, Alkotmány út 2-4, Vácrátót, 2163 Hungary; 3 Evolutionary Ecology Research Group, Institute of Ecology and Botany, Centre for Ecological Research, Alkotmány út 2-4, Vácrátót, 2163 Hungary; 4 Department of Ecology, University of Szeged, Közép Fasor 52, Szeged, 6726 Hungary; 5 Institute of Aquatic Ecology, Centre for Ecological Research, Karolina út 29, Budapest, 1113 Hungary; 6 Senckenberg Deutsches Entomologisches Institut, Eberswalder Str. 90, 15374 Müncheberg, Germany; 7 MTA-DE Biodiversity and Ecosystem Services Research Group, Egyetem tér 1, Debrecen, 4032 Hungary; 8 Department of Ecology, Faculty of Science and Technology, University of Debrecen, Egyetem tér 1, Debrecen, 4032 Hungary

**Keywords:** Biodiversity, Community ecology, Grassland ecology, Ecology, Ecology

## Abstract

Fragmented natural habitats within human-transformed landscapes play a key role in preserving biodiversity. Ants as keystone species are essential elements of terrestrial ecosystems; thus, it is important to understand the factors influencing their presence. In a large-scale multi-site study, we surveyed ant assemblages using sweep netting and D-vac sampling on 158 ancient burial mounds preserving grassland habitats in agricultural landscapes in East-Hungary. We asked the following questions: (1) How do habitat factors and landscape composition affect species richness and functional diversity of ants? (2) Which ant traits are affected by habitat factors and landscape composition? Despite their small sizes, mounds as permanent and relatively undisturbed landscape elements could provide safe havens for diverse ant assemblages even in transformed agricultural landscapes. The complex habitat structure of wooded mounds supported high species and functional diversity of ant assemblages. Ant species on wooded mounds had small or medium-sized colonies, enabling the co-existence of more species. The effect of landscape composition on ant assemblages was mediated by habitat factors: steep slopes buffered the negative effect of the cropland matrix and enabled higher ant diversity.

## Introduction

Habitat loss and fragmentation pose a major threat to biodiversity worldwide^1^. Due to the intensified anthropogenic land transformation activities (e.g., ploughing, forestation and the spread of urban infrastructure), in intensively used landscapes, several natural and semi-natural grassland habitats survived only in small fragments, acting as habitat islands^2^. Despite their small size, these terrestrial islands can considerably contribute to the maintenance of grassland biodiversity^3,4^. Species richness and composition of grassland islands are influenced both by abiotic and biotic environmental factors acting at the level of the habitat patch (habitat factors) and the habitats present in the surrounding landscape (landscape factors). Both habitat factors and landscape composition can act as filters for the establishment and persistence of animal and plant species^5,6^. Many arthropod taxa are especially sensitive to the human-induced changes at the habitat or landscape level^7,8^.

Arthropods are important components of biodiversity and provide essential ecosystem services such as pollination^9^, biological pest control^10^ or decomposition^11^. Populations of arthropods in grassland islands might be affected by several abiotic and biotic habitat factors such as habitat area, habitat heterogeneity and vegetation structure^12,13^. In small habitat islands the reduction of intact core areas and increased edge effect can lead to considerable changes in the abiotic environment of the fragments (such as increased temperature, decreased air humidity and soil moisture)^14^. These changes can lead to the extinction of area-sensitive habitat specialist species and may promote the encroachment of generalists^15^. Given their small size, small grassland fragments are generally not managed which even in the short run results in the accumulation of dead plant biomass and the encroachment of woody species^16^. Negative effects of small habitat area can be compensated by abiotic environmental heterogeneity that might support the co-existence of several contrasting microsites even within a small area and by that support the coexistence of species with different environmental needs^17^.

Landscape composition in the surrounding matrix (i.e., characteristics of the surrounding landscape expressed by the type and amount of habitats in the landscape irrespective of their spatial arrangement) can also considerably influence the biodiversity of grassland islands^5,18^. Changes in the landscape composition, such as transformation of grasslands into croplands or tree plantations can exert detrimental effects especially on habitat specialist species; and thus can lead to a reduction in their population sizes^19^, or even to local extinctions^20^. At the same time the increased amount of anthropogenic habitats can increase the species richness and abundance of generalist species in the focal grassland habitat patch by the enhanced rate of immigration originating from the neighbouring areas^5,10^. Such processes have been documented in various taxa including plants, vertebrates and arthropods^12,21,22^.

In agricultural landscapes of Eurasia, besides verges, field margins and midfield islets the ancient burial mounds (also named as ‘kurgans’) represent one of the most widespread grassland fragments that often provide safe havens for several grassland species^3,12,23,24^. Burial mounds constructed by ancient steppic cultures during the Bronze and Iron ages were typically built from soil that was piled upon a round base with a diameter varying from a couple of metres to 100 m, and with a height of 0.5‒15 m^24,25^. Burial mounds are widespread landmarks distributed from Central Europe to East Asia; present days their total number is approximately 500,000^24^. Due to their steep slopes and social taboos associated with them, they have often remained intact from human disturbances such as ploughing, and thus may act as safe havens for grassland species across Eurasia^24^. Their unique hill shape with steep slopes results in the formation of different microhabitats (e.g., slopes with different aspects) characterised by different abiotic parameters such as soil nutrient content and soil moisture^26,27^. Previous studies showed that these abiotic patterns result in the formation of distinct microhabitats^17,28^. Thus, despite their relatively small area, mounds are characterised by a diverse mosaic of microhabitats and a high plant diversity^17,25^. Despite the fauna is an integral part of dry grassland communities preserved by the mounds, most studies focused on their vegetation, and only a few researches dealt with their fauna (but see Marcolin et al.^29^; Deák et al.^12^ and Tóth et al.^26^).

To explore the effects of habitat factors and landscape composition on the fauna of grassland islands, ants (Hymenoptera: Formicidae) are considered as good model taxa, as (i) they are sensitive to habitat loss on the landscape level^30^, and the species composition of ant assemblages can be affected by the area of the focal habitat patch^31^; (ii) ants respond quickly to changes in habitat quality^32^; (iii) they show high diversity in many habitat types^33^; (iv) they play essential role in almost every terrestrial ecosystem type, influencing other species through mutualistic interactions with various hemipterans, plants and fungi^33–36^; and (v) ants interact with soil processes by mediating chemical changes such as the shifting of pH towards neutral values and increasing the nutrient content of soil close to their nests^37^, hence they can be considered as ecosystem engineers^34,38^. Besides the changes in the species richness of ant assemblages, habitat factors and landscape composition can also affect their functional traits in small habitat islands^39^. Based on the inter-taxa differences in habitat requirements^33^, functional grouping of ants might provide a general understanding of the responses of ants to habitat factors and landscape composition^40,41^.

In the present work, we explored the effects of habitat factors (vegetation structure and mound characteristics in the focal patch) and landscape composition (amount of different habitat types in the surrounding landscape) on ant assemblages in a large-scale multi-site study, involving 158 grassland islands. As a model habitat, we used burial mounds covered by semi-natural dry grassland vegetation, situated in agricultural landscapes. We asked the following questions: (1) What are the effects of habitat factors and landscape composition on the species richness, functional diversity and abundance of ant assemblages maintained in habitat islands that are embedded in transformed agricultural landscapes? (2) Which ant traits are affected by habitat factors and landscape composition?

## Results

In total, 19,713 ant specimens belonging to 33 species and 4 subfamilies were collected and identified from the 158 surveyed mounds. The 33 species represented about 26% of the ant fauna of Hungary^42^. Species belonged to subfamilies Myrmicinae (15 species), Formicinae (13), Dolichoderinae (3) and Ponerinae (2). Among the genera found, *Myrmica* (6 species) was represented by the largest number of species, followed by *Formica* (5), *Lasius* (5) and *Temnothorax* (5). Ant species richness ranged from 0 to 12 species and abundance ranged from 0 to 692 individuals per mound. The two most abundant ant species were the grassland specialist *L. bombycina* and grassland-related *Tetramorium* cf. *caespitum* (65.9% and 12.0% of the collected specimens, respectively). Assemblages were characterized by a wide spectrum of habitat preferences, ranging from typical wood-living ants (e.g. *Dolichoderus quadripunctatus*, *Lasius citrinus*) to species typical of open grasslands (e.g. *L. bombycina*, *Formica rufibarbis*) and from species preferring cool and wet habitats (e.g. *Lasius plathythorax*, *Myrmica rubra*) to species preferring warm and dry environments (e.g. *Camponotus atricolor*, *Messor structor*).

**Figure 1:**
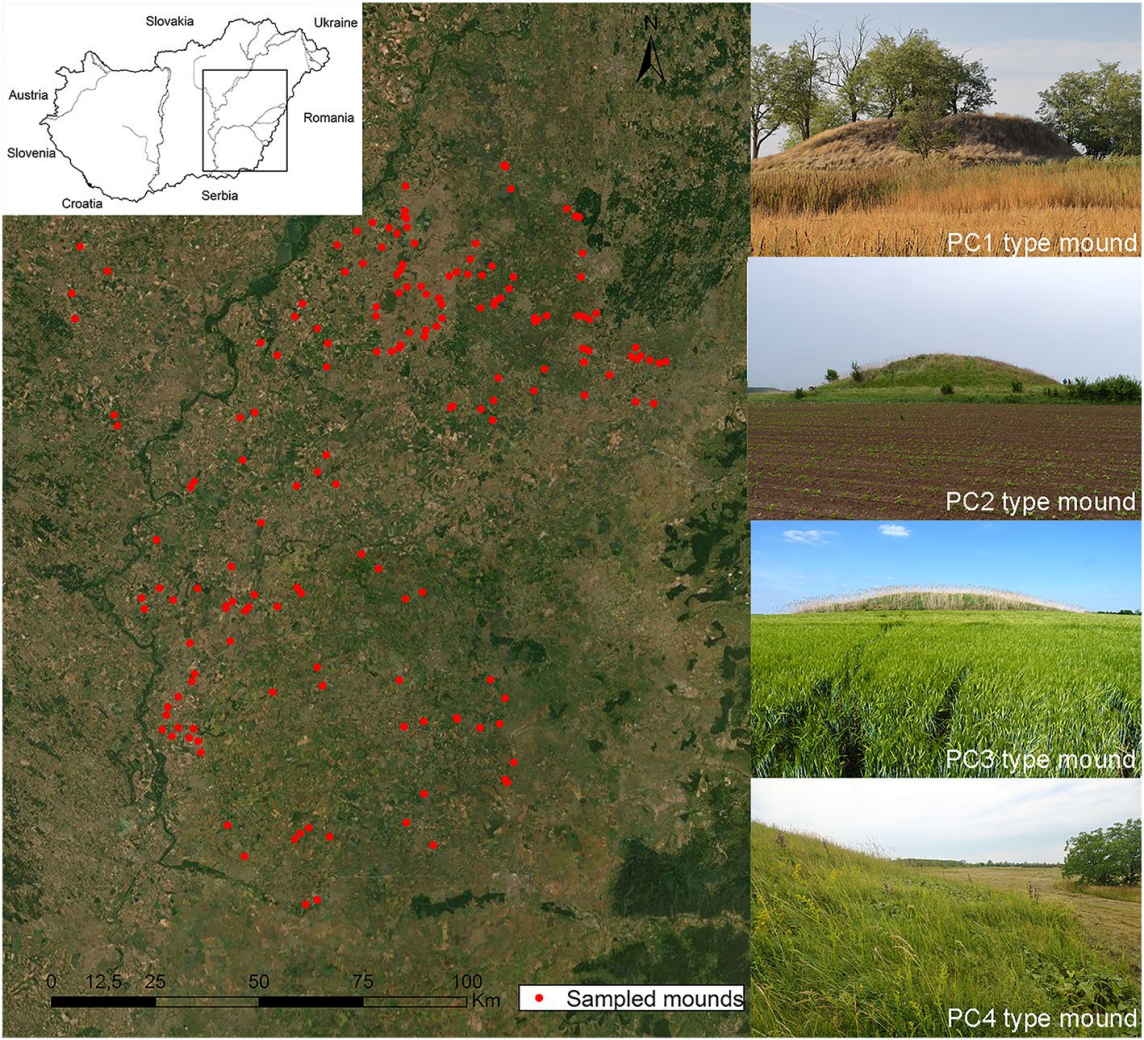
Map of the studied mounds, and photos of the four basic mound types (based on the PCA analysis of the predictors describing the physical properties and vegetation of the mounds and the composition of the neighbouring landscape). The map is based on satellite imagery provided by the ESRI basemap function. The map was created by using the ESRI ARCGIS 10.2 software (ver 2.14.19; http://www.qgis.org). Photographs were taken by B. Deák.

The principal component (PCA) analyses revealed that the surveyed mounds can be characterised by four principal components (PCs) each referring to a combination of original mound predictors (N = 11) involving habitat factors and landscape composition (Table 1, Fig. 1). PC1 corresponded to large mound height, high cover of woody species, thick litter layer and steep slopes. PC1 correlated positively with high amounts of croplands in the neighbouring landscape and negatively with the proportion of grasslands. These attributes are typical to large mounds prone to woody encroachment and embedded into extensive agricultural fields. PC2 corresponded to small mound area and height, high amount of croplands and low amount of forests and wetlands in the buffer zone. These attributes are typical of small mounds in agricultural areas that are too small for the establishment of closed woody vegetation. PC3 corresponded to low cover of woody plants and a high cover of tall herb species on the mounds. It also correlated positively with the amount of wetlands and negatively with the amount of forests in the neighbouring landscape. These attributes are typical of mounds invaded by terrestrial reed (*Phragmites australis*). PC4 corresponded to large mound height, steep slopes, low cover of woody species and low amount of forests in the landscape. These attributes are typical of high mounds covered by dry grassland vegetation.

**Table 1:** Correlation of the studied habitat factors and landscape composition with the four PCs explaining 60.58% of variation in total.

	PC1	PC2	PC3	PC4
Proportion of explained variance	0.21	0.16	0.13	0.11
Eigenvalue	2.31	1.72	1.41	1.23
**Habitat factors**				
Mound area	− 0.07	**− 0.57**	0.03	0.03
Mound height	**0.23**	**− 0.56**	0.05	**0.29**
Mean slope inclination	**0.27**	− 0.11	− 0.16	**0.57**
Mean litter thickness	**0.24**	− 0.14	0.12	− 0.03
Mean vegetation height of herbaceous species	**0.26**	− 0.02	**0.41**	**− 0.46**
Cover of herbaceous plants	0.12	− 0.12	**0.59**	− 0.12
Cover of woody plants	**0.31**	− 0.12	**− 0.44**	**− 0.35**
**Landscape composition**				
Percentage of croplands	**0.52**	**0.30**	0.06	0.17
Percentage of forests	0.09	**− 0.33**	**− 0.39**	**− 0.41**
Percentage of grasslands	**− 0.57**	− 0.11	0.02	0.00
Percentage of wetlands	− 0.08	**− 0.26**	**0.26**	0.06

Significant correlations are marked with boldface.

High model weights and positive model-averaged parameter estimates indicated that high PC1 values positively affected Shannon and Rao diversity of ant assemblages (Table 2). It also supported forest-related and forest specialist ants, ants preferring dry habitat conditions and having small colony size. High PC2 values correlated negatively with total ant species richness. High values of PC3 negatively affected ant species confined to cool habitats and forests and supported ants with narrow habitat preference. Rao diversity of ant assemblages was low on mounds characterised by high PC3 values. PC4 negatively correlated with total species richness of ants, and positively affected ants confined to dry habitats.

**Table 2:** Results of the General Linear Mixed Models fitted on the species richness, Shannon diversity, Rao diversity and functional traits.

	PC1		PC2		PC3		PC4	
	ω	θ	ω	θ	ω	θ	ω	θ
Species richness	0.308	0.006	0.670	− 0.037	0.351	− 0.009	0.508	− 0.024
Shannon diversity	0.343	0.024	0.055	− 0.002	0.089	− 0.004	0.026	< 0.001
Rao diversity	0.200	0.026	0.151	− 0.016	0.312	− 0.048	0.069	< 0.001
Colony size	0.812	− 0.065	0.034	0.001	0.043	0.001	0.027	< 0.001
Habitat preference	0.388	0.024	0.074	− 0.003	0.768	− 0.062	0.036	− 0.001
Humidity preference	0.247	− 0.012	0.018	< 0.001	0.020	< 0.001	0.107	− 0.005
Temperature preference	0.015	< 0.001	0.025	< 0.001	0.129	− 0.005	0.047	− 0.001
Plasticity	0.046	0.001	0.022	< 0.001	0.148	− 0.007	0.020	< 0.001
Behaviour	0.015	< 0.001	0.016	< 0.001	0.017	< 0.001	0.017	< 0.001

Notations: *PC* principal component, *ω* sum of model weights, *θ* model-averaged parameter estimates.

Wooded mounds (characterised by high PC1 values) were preferred by several forest specialist and forest-related (e.g. *Formica truncorum*, *Temnothorax crassispinus*) and generalist (e.g. *Formica fusca*, *Ponera coarctata*) species (Supplementary Figure S1). We also observed grassland specialist and grassland-related species (e.g. *M. structor*, *Myrmica schencki*) in small numbers on these mounds. We detected several grassland specialist and grassland-related ant species (e.g. *Myrmica curvithorax*, *Ponera testacea*) and also some forest specialist and forest-related ant species (e.g. *L. citrinus*, *L. platythorax*) on mounds without woody vegetation.

## Discussion

Based on a survey of 158 mounds, our results showed that the species richness, Shannon diversity, functional traits and functional diversity of ant assemblages in grassland habitat islands were affected by the different combinations of habitat factors and landscape composition. We found high Shannon diversity of ants on woody mounds with steep slope inclination and thick litter layer. By providing high vertical structural complexity the presence of woody species on these mounds could increase the diversity of microsites (e.g. dead and decayed branches and detached barks) used by different ant species^43^. Steep mound slopes can also contribute to the heterogeneity of microsites within the mounds because steep slopes with different aspects are characterised by contrasting environmental conditions (solar radiation, microclimates and soil properties) (see also Bátori et al.^40,44^). As the steepness of the slope can increase the contrasts among the microsites by influencing the amount of solar radiation received and precipitation runoff, between-microhabitat differences are larger in mounds with steeper slopes^17^. The presence of contrasting microhabitats even within a range of a couple of meters allows the co-existence of ant species with different environmental requirements that can positively affect the Shannon diversity of ant assemblages^17,24,40^. On mounds characterised by high PC1 values litter accumulation can also be beneficial for several ant species and might increase ant diversity because it can provide both shelter and foraging sites for them^45^.

The high availability and variability of nesting and foraging sites provided by woody plants favoured forest specialist and forest-related species which build their nests in decayed or fallen tree branches and forage on trees (*D. quadripunctatus, L. fuliginosus*, *Temnothorax* spp.)^46^. Despite that woody habitats generally support ants preferring moist habitats ants related to dry habitats were typical on woody mounds. This is probably due to that the studied mounds were predominantly covered by black locust (*Robinia pseudoacacia*) characterised by a low canopy cover that allows a high level of solar radiation even on the soil surface^47,48^. Thus, it results in dry habitat conditions that favour the populations of xerophilous ant species.

We found that woody mounds, which had high environmental heterogeneity, were characterized by species with large body size and small or medium sized colonies. Smaller sized but more diverse habitat patches favour opportunistic species, which usually have smaller colony sizes, and therefore smaller demand for food and smaller foraging areas, allowing the coexistence of many species sharing these resources. Species with large body size were represented either by habitat generalist ants (e.g., *F. fusca*) or species associated with woodland habitats (e.g., *L. fuliginosus*, *F. truncorum*)^35^. Due to their large body size, workers of these species are able to forage within considerable distances on the ground and on trees and exploit food sources fast and efficiently. High niche diversity was also indicated by the high functional diversity of ant assemblages on woody mounds. In contrast to woody mounds characterised by high PC1 values, mounds invaded by terrestrial reed (high PC3 value) and the ones with steep slopes and short grassland vegetation (high PC4 value) harboured fewer ant species. The lack of vertical environmental heterogeneity and additional resources provided by woody species resulted in a reduced number of niches leading to a reduction of co-occurring species on these mounds.

Mounds with steep slopes provide a fine-scale heterogeneity of microhabitats for plants and increases their mound-level diversity^17,24^. Here we found that for ants, the heterogeneity introduced by woody species is more important than topographic heterogeneity itself, as higher ant diversity was found on mounds with steep slopes and woody species than on woodless ones. Woodless mounds provide fewer ecological niches with less resources and nesting sites for ants, which is indicated by the presence of fewer ant species. Due to the lack of moderate shading provided by woody vegetation these mounds can be considered as extremely warm habitats supporting few ant species related to warm open habitats.

Degraded mounds encroached by terrestrial reed were mostly populated with few small generalist species with various habitat preferences such as *Temnothorax albipennis*, *P. testacea* and *Myrmecina graminicola*. Based on our findings, functional diversity of ant assemblages was also low in such mounds likely due to the low environmental complexity and the homogenous degraded vegetation mostly represented by reed. The lack of shading and the windshield effect of the tall, reed dominated vegetation likely resulted in a warm microclimate. It explains why these mounds were characterized by stenotopic and oligotopic species associated with warm grassland habitats. This is also supported by the presence of small grassland specialist species (e.g. *P. testacea* and *M. curvithorax*).

Our results suggest that the effect of landscape composition on the ant assemblages was mediated by the habitat factors. The studied mounds were located in an agricultural landscape, where the dominant land cover type was cropland (55% proportion), which land cover type is in general a hostile habitat for ant species^49^. Mounds with high PC1 and PC2 values situated in landscapes with the highest amount of croplands. Interestingly we found, that despite the large amount of neighbouring croplands, the Shannon diversity of ants was large on mounds with a high PC1 value; however, ant species richness was small on mounds with high PC2 values. A possible explanation is that mounds with high PC2 values were flat, while mounds with high PC1 values were high and had steep slopes, that can mitigate several negative effects received from the cropland matrix, such as fertilization, that can reduce ant diversity^50^. Steep slopes form a physical barrier against agricultural activities^24^, and also create a high level of environmental heterogeneity that can support the coexistence of several species^17,51^. This suggests that habitat islands embedded in croplands, but having steep slopes can support diverse ant assemblages by buffering negative effects received from the surrounding matrix and by creating environmental heterogeneity.

### Conservation outlook

Our results showed that despite their small sizes (less than one hectare), ancient burial mounds can provide important habitats for ant species even in transformed agricultural landscapes. Similar results were found by Azcarate et al.^39^ who studied analogous features, gypsum outcrops in a Spanish agricultural landscape and found that these permanent and relatively undisturbed small habitat islands are important safe havens for diverse ant assemblages. The large level of environmental heterogeneity and habitat complexity provided by mounds covered by woody vegetation supported the existence of diverse ant assemblages. However, ant species on woody mounds were mainly generalists and species typical of forests. Clearly, diversity of these species can play an important role in maintaining agro-biodiversity in agricultural landscapes. However, from a conservation viewpoint, the situation is more complex and beyond the measures of diversity in a general sense. Since originally mounds of the steppe and forest steppe zone were typically covered by short grasslands, the presence of woody species can be considered as a habitat degradation process. Thus, in a conservation sense, mounds holding grassland vegetation and ants confined to grassland vegetation can be considered as of a top priority. As also shown by our results, these mounds had a low ant species richness with species related to warm open habitats such as dry grasslands. Thus, conservation efforts should consider the maintenance of mounds with grassy open habitats even by cutting woody vegetation, since mounds covered by grasslands have the highest potential to preserve grassland related ant species. However, in order to maintain ant diversity on the mounds it can be considered to keep individual trees or small patches of native woody vegetation that might sustain the populations of forest related ants. Similarly, in the forest-grassland ecotone in South Brazil, it was found that low number of trees can considerably increase ant diversity in grasslands^43^.

## Materials and methods

### Study area

The study area is located in the Great Hungarian Plain (East Hungary) and characterised by a continental climate with cold winters and warm summers. The mean annual precipitation is 538 mm and the mean annual temperature is 10.4 °C^52^. The historical landscape of the study area was predominantly characterised by open habitats, such as steppes, forest steppes and wetlands. However, during the past centuries many of the natural habitats have been lost, since they have been converted into intensive agricultural fields and urban areas since the eighteenth century^22^. Present days the remaining extensive dry steppe grasslands are situated exclusively inside the few protected areas. In non-protected agricultural landscapes covering vast majority of the region dry grasslands typically exist in areas inadequate for agricultural production. They are generally small in size and surrounded by anthropogenic habitats such as croplands or tree plantations^3^. Steppe grasslands of the study area are predominantly represented by loess and alkaline grasslands. Loess grasslands are characterised by short grass species such as *Festuca rupicola, Koeleria cristata* and *Poa angustifolia*, and harbour a high diversity of forb species like *Achillea collina*, *Filipendula vulgaris, Fragaria viridis*, *Galium verum*, *Salvia* spp., *Trifolium* spp. and *Verbascum phoeniceum*. Alkaline grasslands are also characterised by a short vegetation. Their typical grass species is *Festuca pseudovina*, and the dominant forb species are *Achillea setacea*, *Artemisia santonica*, *Limonium gmelinii* and *Scorzonera cana*. In the deeper lying areas mesic hay meadows, reed beds and marshes consisted by *Typha latifolia* and *T. angustifolia* are present. Natural forest stands have almost completely disappeared from the region and were replaced by alien poplar (*Populus* × *canadensis*) and black locust (*Robinia pseudoacacia*) plantations. Spontaneous stands of adventive woody species such as *Acer negundo*, *Fraxinus pennsylvanica* and *R. pseudoacacia* are also typical of the study area. In the extensive agricultural fields, the most typical crops are annual ones, such as cereal, corn and sunflower. These characteristics make our study area a good example for transformed European lowland landscapes.

### Field data collection

A total of 158 mounds harbouring dry grassland vegetation were selected for the study (Fig. 1). The surveyed mounds were characterised by various landscape composition regarding the amount of grasslands, forests, wetlands and croplands (Supplementary Table S1). The mounds were surveyed between May and June in three consecutive years (2014–2016) with each mound surveyed once. During the survey we collected information on habitat factors, i.e., those physical attributes and vegetation parameters of the mounds that might affect ant assemblages on the local habitat level. On each mound we measured the inclination of north-, east-, south- and west-facing slopes with an inclinometer, and for the calculations we used the mean of the four degree scores. Total cover of herbaceous plants and woody species were recorded by visual estimation; we estimated the percentage cover of each plant species present on the mound and summed their cover scores. This way we got a nuanced picture on the vegetation structure. We measured the mean litter thickness and mean height of herbaceous vegetation at one randomly chosen sampling point on the top of the mounds and in the midpoint of the north-, east-, south- and west-facing slopes. For the calculations we used the mean of the five scores.

Sweep-net and D-vac sampling methods were used to collect ants along four transects positioned according to the four cardinal directions (north, east, south and west) from the bottom to the top of the mounds. The combination of the two sampling methods enabled to collect ants with different lifestyles and environmental preferences and thus ensured that all kinds of taxonomic and functional groups were collected during the study. By D-vac, species foraging on and directly below (cryptobiotic species like *Ponera* spp.) the soil surface can be collected, while sweep-netting is a proper method for collecting species above the soil surface, foraging on the vegetation. Sweep-net samples were collected applying 50 sweeps by a 40 cm diameter sweep net in each transect. All individuals were transferred to a labelled plastic bag. The D-vac with a 12-cm-diameter sampling cone and collecting bag was placed 15 times (held above the soil surface for 5 s/placement) along each transect. After 15 placements, the collecting bag was removed from the D-vac, and the collected individuals were also stored in a labelled plastic bag. In the lab, ants were sorted from the samples and stored in 70% ethanol until their identification at species level using standard keys^53,54^. For the analyses we used samples pooled on the mound level.

### Ant traits

For the trait-based analyses of ant assemblages, we assigned the following functional traits to the recorded ant species: colony size (10; 100; 1,000; 10,000; 100,000 individuals), habitat preference [grassland specialist species (species occurring almost exclusively in grassland habitats); grassland-related species (species occurring mostly in grassland habitats); generalists (species with no habitat preferences); forest-related species (species occurring mostly in woodlands); forest specialist species (species occurring almost exclusively in closed woodland habitats)], humidity requirement [xerophiles (species occurring almost exclusively in dry habitats); meso-xerophiles (species occurring mostly in dry habitats); mesophiles (species occurring in habitats with moderate humidity); meso-hygrophiles (species occurring mostly in moist habitats); hygrophiles (species occurring almost exclusively in moist habitats)], temperature requirement [thermophiles (species occurring almost exclusively in warm habitats); thermo/mesothermophiles (species occurring mostly in warm habitats); mesothermophiles (species occurring in moderately warm habitats); meso/oligothermophiles (species occurring mostly in cool habitats); oligothermophiles (species occurring almost exclusively in cool habitats)], habitat plasticity [stenotopic species (species occurring in one type of habitat, e.g., xerothermic grasslands); oligotopic species (species occurring in habitats of a few similar types, e.g., in various deciduous forests, or species requiring a specific habitat factor, e.g., a certain level of humidity); polytopic species (species occurring in many different habitats within their definite category, e.g., in various woodland habitats); eurytopic species (species which can live both in woodland and grassland habitats, and show no distinct preference for any type of habitat or habitat factor)] and behaviour [submissive (species that avoid conflict with workers of other colonies or species, except when defending their own nest); intermediate (species that are aggressive when defending or trying to take over food sources); aggressive (species that are very combative and assertive to workers of other colonies or species)] based on the descriptions of Czechowski et al.^54^ and Seifert^53^ (Supplementary Table S2).

### GIS processing of landscape level data

We calculated the area and height of each mound using 1:10 000 topographic maps^55^. To characterise the landscape composition around the mounds, we used the data provided by the multi-level National Ecosystem Map of Hungary (NEMH; data source: Ministry of Agriculture^56^). The NEMH was compiled by using thematic GIS layers (e.g. detailed vegetation and habitat maps, soil maps, maps of the national forestry database and land use data from the Land Parcel Identification System) providing data about typical land cover and habitat types at a spatial resolution of 20 m. For data validation, we used our own habitat maps and ortophotos of the surrounding areas. Taking into account the relatively limited foraging range of ants^33,57^, we investigated the landscape composition in buffer zones with 50 m radius around each surveyed mound. Using the data from the NEMH we assigned all habitat types occurring in the 50 m buffer zones into six main habitat categories: urban areas, croplands, grasslands, forests, wetlands and open water. Using QGIS 3.10.8 LTR (http://qgis.osgeo.org)^58^ we calculated the relative percentage cover of each habitat category in a 50 m buffer zone around all mounds. Since the ‘open water’ category was highly underrepresented in the buffers (mean cover 0.1%, relative frequency 3.6%) we omitted it from our study in order to reduce the number of factors used for the analyses. Even though the mean cover and relative frequency of ‘urban habitats’ (8% and 45.8% respectively) were higher compared to open water we also excluded it from the analyses: this category in the NEMH is composed by very diverse and contrasting land cover categories including extensively used farm yards, urban greenspaces and built concrete surfaces, that cannot be treated as a homogeneous land cover type in an ecological sense.

### Statistical analysis

As our dataset included a large number of variables related to habitat factors and landscape composition, first we conducted a Principal Components Analysis which aims to reduce the number of original predictors (Supplementary Table S1) into a small set of independent variables (principal components; PC). Thus, using PCs with eigenvalues larger than 1.0, we were able to reduce the N = 11 original predictors into a set of four PCs explaining 60.58% of the total variance, which we added to the mound dataset and refer to mound predictors henceforth. The relationships among original and PC mound predictors are described in Supplementary Table S3. We also calculated the variance inflation factors between all original predictors to test for multicollinearity using the ‘faraway’ package in R^59^. VIF for ten of the eleven original predictors was between 1 and 5, thus can be considered as moderately correlated, we observed a high value (6.4) for the percentage of croplands in the 50 m buffer of the mounds that suggests a stronger correlation (Supplementary Table S4).

Next, we merged the mound dataset and the ant dataset using the mound ID-s. In the following step, we calculated species richness and Shannon diversity of ant assemblages for each mound, applying the ‘vegan’ R-package^60^. To calculate Rao diversity and community-weighted mean (CWM) trait values for each ant trait (colony size, habitat affinity, humidity and temperature requirements as well as plasticity and behaviour) for the ant assemblages in each mound, we used the dbFD function available in the ‘FD’ package^61^. Thus, finally we obtained ten response variables (species richness, Shannon-diversity and Rao diversity as well as CWM-values of ant traits), which we refer to as ant assemblage responses (Supplementary Table S5).

Finally, to identify associations among ant assemblage responses and the four mound predictors (PCs), we fitted General Linear Mixed Models (GLMM) for each response as a function of all mound predictors, using region as random factor to control for possible spatial autocorrelation. For species richness data we used Poisson distribution and untransformed data, for all other variables we used a model with normal distribution. In the following step, we conducted model selection on the full set of all possible models. To assess the relative importance of principal components in explaining the variance of ant assemblage responses, we computed model-averaged parameter estimates and sums of model weights^62,63^. We considered a principal component to be influential in explaining the variance in a particular response variable, if it had a high model weight value. The direction of its effect was provided by the positive or negative sign of the model-averaged parameter estimates. All statistical analyses have been performed within the R statistical programming environment^64^.

## Supplementary Information

Supplementary Information.

## Data Availability

The datasets generated during and/or analysed during the current study are available from the corresponding author on reasonable request.
