# Supplementary material for: Functional composition of ant assemblages in habitat islands is driven by habitat factors and landscape composition
Source: Sci Rep. 2021 Oct 25;11:20962. doi: 10.1038/s41598-021-00385-5 (PMC8546063; doi:10.1038/s41598-021-00385-5)

Deák, B. & Báthori, F., Lőrinczi, G., Végvári, Z., Nagy D., D., Mizser, S., Torma, A., Valkó, O. & Tóthmérész, B. Functional composition of ant assemblages in habitat islands is driven by local factors and landscape composition.

Supplementary Table S1. Minimum, maximum, mean values of the studied original predictors.

Original predictor	min	max	mean	SD
Cover of herbaceous plants (%)	56.6	166.9	104.9	21.8
Cover of woody plants (%)	0	98.3	20.5	26.7
Mean litter thickness (cm)	0	70	2.4	9.0
Mean slope inclination (◦)	4	30	13.5	5.4
Mean vegetation height of herbaceous species (cm)	11	159	65.2	27.6
Mound area (m^2)	84	7097	2227.3	1536.2
Mound height (m)	1	12	5.0	2.5
Percentage of croplands around the mounds (%)	0	100	55.0	36.8
Percentage of forests around the mounds (%)	0	67.5	6.1	13.5
Percentage of grasslands around the mounds (%)	0	100	21.6	32.8
Percentage of wetlands around the mounds (%)	0	69.7	8.3	14.1

Supplementary Table S2. Species list and traits of the studied ant species.

Subfamily	Tribe	Species	Colony size	Habitat preference	Humidity requirement	Temperature requirement	Habitat plasticity	Behaviour
Formicinae	Camponotini	*Camponotus atricolor*	100	grassland specialist	xerophile	thermophile	stenotopic	intermediate
Dolichoderinae	Dolichoderini	*Dolichoderus quadripunctatus*	100	forest specialist	mesophile	thermo/mesothermophile	oligotopic	submissive
Formicinae	Formicini	*Formica cunicularia*	1000	grassland related	meso-xerophile	thermo/mesothermophile	polytopic	submissive
Formicinae	Formicini	*Formica fusca*	1000	generalist	mesophile	mesothermophile	eurytopic	submissive
Formicinae	Formicini	*Formica rufibarbis*	100	grassland specialist	meso-xerophile	thermo/mesothermophile	oligotopic	intermediate
Formicinae	Formicini	*Formica sanguinea*	1000	generalist	meso-xerophile	thermo/mesothermophile	polytopic	aggressive
Formicinae	Formicini	*Formica truncorum*	10000	forest related	mesophile	mesothermophile	oligotopic	aggressive
Formicinae	Lasiini	*Lasius bombycina*	10000	grassland specialist	mesophile	thermo/mesothermophile	oligotopic	intermediate
Formicinae	Lasiini	*Lasius citrinus*	1000	forest specialist	meso-xerophile	thermo/mesothermophile	oligotopic	submissive
Formicinae	Lasiini	*Lasius fuliginosus*	100000	forest related	mesophile	mesothermophile	oligotopic	aggressive
Formicinae	Lasiini	*Lasius niger*	1000	grassland related	mesophile	mesothermophile	polytopic	aggressive
Formicinae	Lasiini	*Lasius platythorax*	1000	forest related	mesophile	meso/oligothermophile	polytopic	aggressive
Myrmicinae	Stenammini	*Messor structor*	10000	grassland specialist	xerophile	thermophile	stenotopic	intermediate
Myrmicinae	Crematogastrini	*Myrmecina graminicola*	10	generalist	mesophile	thermo/mesothermophile	oligotopic	submissive
Myrmicinae	Myrmicini	*Myrmica curvithorax*	100	grassland specialist	meso-xerophile	thermophile	oligotopic	intermediate
Myrmicinae	Myrmicini	*Myrmica rubra*	1000	generalist	meso-hygrophile	meso/oligothermophile	eurytopic	aggressive
Myrmicinae	Myrmicini	*Myrmica sabuleti*	100	generalist	meso-xerophile	thermo/mesothermophile	oligotopic	intermediate
Myrmicinae	Myrmicini	*Myrmica scabrinodis*	100	generalist	mesophile	mesothermophile	polytopic	submissive
Myrmicinae	Myrmicini	*Myrmica schencki*	100	grassland specialist	meso-xerophile	thermo/mesothermophile	oligotopic	intermediate
Myrmicinae	Myrmicini	*Myrmica specioides*	100	grassland specialist	meso-xerophile	thermophile	oligotopic	aggressive
Formicinae	Plagiolepidini	*Plagiolepis pygmaea*	10000	grassland related	xerophile	thermophile	oligotopic	intermediate
Formicinae	Plagiolepidini	*Plagiolepis taurica*	10000	grassland related	xerophile	thermophile	oligotopic	intermediate
Ponerinae	Ponerini	*Ponera coarctata*	10	generalist	meso-xerophile	thermo/mesothermophile	oligotopic	intermediate
Ponerinae	Ponerini	*Ponera testacea*	10	grassland specialist	xerophile	thermophile	stenotopic	intermediate
Myrmicinae	Solenopsidini	*Solenopsis fugax*	100000	grassland specialist	meso-xerophile	thermophile	oligotopic	aggressive
Dolichoderinae	Tapinomini	*Tapinoma erraticum*	1000	grassland specialist	xerophile	thermophile	stenotopic	submissive
Dolichoderinae	Tapinomini	*Tapinoma madeirense*	1000	grassland specialist	xerophile	thermophile	stenotopic	submissive
Myrmicinae	Crematogastrini	*Temnothorax affinis*	100	generalist	meso-xerophile	thermophile	stenotopic	submissive
Myrmicinae	Crematogastrini	*Temnothorax albipennis*	10	generalist	meso-xerophile	thermo/mesothermophile	stenotopic	submissive
Myrmicinae	Crematogastrini	*Temnothorax crassispinus*	10	forest specialist	mesophile	mesothermophile	oligotopic	submissive
Myrmicinae	Crematogastrini	*Temnothorax nigriceps*	10	grassland related	meso-xerophile	thermophile	stenotopic	submissive
Myrmicinae	Crematogastrini	*Temnothorax unifasciatus*	100	generalist	meso-xerophile	thermo/mesothermophile	oligotopic	submissive
Myrmicinae	Crematogastrini	*Tetramorium* cf. *caespitum*	1000	grassland related	meso-xerophile	thermo/mesothermophile	polytopic	aggressive

Supplementary Table S3. Relationships among predictors and principal component variables. Asterisks denote significant differences ($p \leq 0.05$).

Principal Component	Predictor	p value	Sig.
PC1	Cover of herbaceous plants	0.013	*
PC1	Mound area	0.139	
PC1	Mound height	≤0.001	*
PC1	Mean slope inclination	≤0.001	*
PC1	Cover of woody plants	≤0.001	*
PC1	Mean litter thickness	≤0.001	*
PC1	Mean vegetation height	≤0.001	*
PC1	Percentage of croplands around the mounds	≤0.001	*
PC1	Percentage of grasslands around the mounds	≤0.001	*
PC1	Percentage of forests around the mounds	0.050	
PC1	Percentage of wetlands around the mounds	0.085	
PC2	Cover of herbaceous plants	0.035	*
PC2	Mound area	≤0.001	*
PC2	Mound height	≤0.001	*
PC2	Mean slope inclination	0.061	
PC2	Cover of woody plants	0.038	*
PC2	Mean litter thickness	0.015	*
PC2	Mean vegetation height	0.680	
PC2	Percentage of croplands around the mounds	≤0.001	*
PC2	Percentage of grasslands around the mounds	0.048	*
PC2	Percentage of forests around the mounds	≤0.001	*
PC2	Percentage of wetlands around the mounds	≤0.001	*
PC3	Cover of herbaceous plants	≤0.001	*
PC3	Mound area	0.558	
PC3	Mound height	0.434	
PC3	Mean slope inclination	0.010	*
PC3	Cover of woody plants	≤0.001	*
PC3	Mean litter thickness	0.058	
PC3	Mean vegetation height	≤0.001	*
PC3	Percentage of croplands around the mounds	0.298	
PC3	Percentage of grasslands around the mounds	0.739	
PC3	Percentage of forests around the mounds	≤0.001	*
PC3	Percentage of wetlands around the mounds	≤0.001	*
PC4	Cover of herbaceous plants	0.071	
PC4	Mound area	0.595	
PC4	Mound height	≤0.001	*
PC4	Mean slope inclination	≤0.001	*
PC4	Cover of woody plants	≤0.001	*
PC4	Mean litter thickness	0.604	
PC4	Mean vegetation height	≤0.001	*
PC4	Percentage of croplands around the mounds	0.014	*
PC4	Percentage of grasslands around the mounds	0.958	
PC4	Percentage of forests around the mounds	≤0.001	*
PC4	Percentage of wetlands around the mounds	0.389	
PC4	Cover of herbaceous plants	0.071	
PC4	Mound area	0.595	
PC4	Mound height	≤0.001	*
PC4	Mean slope inclination	≤0.001	*
PC4	Cover of woody plants	≤0.001	*

Principal Component	Predictor	p value	Sig.
PC4	Mean litter thickness	0.604	
PC4	Mean vegetation height	≤0.001	*
PC4	Percentage of croplands around the mounds	0.014	*
PC4	Percentage of grasslands around the mounds	0.958	
PC4	Percentage of forests around the mounds	≤0.001	*
PC4	Percentage of wetlands around the mounds	0.389	
PC4	Percentage of wetlands around the mounds	0.389	

Supplementary Table S4. Minimum, maximum, mean values of the studied response variables.

Mound ID	PC1	PC2	PC3	PC4	Species richness	Shannon diversity	Rao diversity	Colony size	Habitat pref.	Humidity requ.	Temp. requ.	Plasticity	Behaviour
1	-2.200	-0.280	0.669	0.081	8.000	0.780	1.112	2.106	0.877	1.915	1.796	1.843	1.830
2	0.547	-1.154	1.248	0.623	8.000	0.780	1.202	2.712	0.161	1.900	1.115	1.144	1.088
3	0.171	-1.651	2.466	-0.289	10.000	1.173	1.831	2.625	0.352	1.844	1.164	1.305	1.180
4	0.249	-0.632	0.170	0.651	8.000	1.028	2.281	2.868	0.355	1.415	0.774	1.111	1.094
5	0.930	-4.018	-1.334	-0.942	5.000	1.307	3.016	2.500	0.560	1.620	1.220	1.420	1.300
6	-2.920	0.202	-0.600	0.614	2.000	0.683	1.854	2.571	0.429	1.571	1.000	1.429	0.571
7	1.269	-0.801	-0.449	0.944	5.000	1.027	2.123	2.186	0.721	1.837	1.605	1.698	1.535
8	-3.448	-0.223	-0.703	0.016	6.000	0.164	0.192	2.973	0.015	1.969	0.988	1.012	1.012
9	-0.431	0.286	1.044	-1.076	7.000	0.793	1.362	2.725	0.275	1.788	1.000	1.175	1.088
10	-0.078	0.020	-0.735	-1.541	11.000	0.820	1.179	2.808	0.167	1.857	1.012	1.106	1.053
11	1.839	0.230	-1.591	-0.027	10.000	0.934	1.497	2.691	0.248	1.764	0.964	1.073	0.952
12	1.499	0.400	-0.585	1.083	10.000	1.360	2.438	2.460	0.532	1.500	0.927	1.194	1.113
13	0.622	-1.523	1.411	0.751	6.000	1.486	4.196	1.875	0.281	0.969	0.688	0.844	1.125
14	0.969	-0.841	-0.977	-0.734	6.000	1.068	2.166	2.647	0.275	1.588	0.902	1.137	0.922
15	2.217	-1.538	-0.383	-1.196	10.000	1.671	3.608	1.983	1.033	1.567	1.367	1.683	0.817
16	-0.050	0.719	-1.800	0.976	5.000	1.062	1.711	2.565	0.348	1.870	1.261	1.348	1.261
17	0.141	1.212	0.011	0.557	7.000	1.569	3.896	1.778	0.778	0.741	0.630	0.741	0.481
18	0.961	-0.875	0.148	0.513	6.000	0.901	2.351	1.608	1.341	1.932	1.648	1.688	0.341
19	1.071	0.740	-1.637	1.397	9.000	0.962	1.462	2.714	0.143	1.786	0.986	1.114	0.986
20	0.026	-2.796	1.287	1.926	7.000	1.210	2.285	2.506	0.339	1.780	1.161	1.244	1.149
21	1.240	0.040	-0.415	1.072	5.000	0.949	1.502	2.646	0.354	1.795	1.134	1.339	1.291
22	-0.581	-1.042	1.002	1.042	6.000	0.733	1.179	2.744	0.223	1.787	1.027	1.218	1.202
23	0.585	-0.091	-1.438	-0.748	5.000	1.316	2.660	2.291	0.636	1.782	1.236	1.418	0.982
24	-0.588	1.182	-0.361	0.631	11.000	1.673	5.330	1.965	1.472	2.161	1.965	2.166	1.513
25	0.657	1.829	-0.337	1.356	4.000	0.968	1.574	2.674	0.326	1.767	1.093	1.326	1.186
26	1.003	-1.971	-1.973	-0.661	10.000	0.925	1.367	2.691	0.218	1.764	0.973	1.100	1.091
27	-2.464	-0.099	1.071	-0.763	4.000	0.330	0.451	2.907	0.047	1.930	1.000	1.047	1.000
28	-2.618	-0.776	-0.438	1.113	4.000	0.689	1.454	2.750	0.125	1.750	0.938	1.063	1.000
29	-1.621	-0.590	0.766	0.782	5.000	0.817	1.875	2.612	0.112	1.638	0.879	1.000	1.112
30	-2.854	0.135	-0.267	0.400	2.000	0.084	0.097	2.984	0.016	1.984	1.000	1.016	1.016
31	-2.024	-1.444	-0.974	-0.721	5.000	0.391	0.685	2.879	0.043	1.890	0.972	1.014	0.993

Mound ID	PC1	PC2	PC3	PC4	Species richness	Shannon diversity	Rao diversity	Colony size	Habitat pref.	Humidity requ.	Temp. requ.	Plasticity	Behaviour
32	0.410	0.497	0.403	1.514	6.000	1.384	3.812	1.966	0.379	1.552	0.862	0.897	1.000
33	0.500	0.413	1.109	0.354	8.000	1.563	4.780	1.929	0.619	1.238	1.119	1.333	1.310
34	1.529	1.070	1.721	-0.332	2.000	0.257	0.371	2.071	0.929	2.000	1.929	1.929	1.929
35	-1.765	-2.132	0.364	1.166	5.000	1.017	1.955	2.627	0.333	1.784	1.137	1.314	1.020
36	-3.059	0.624	0.487	-0.305	6.000	0.388	0.514	2.952	0.056	1.920	0.984	1.048	1.048
37	-2.212	-0.049	0.735	-0.251	5.000	0.768	1.435	2.689	0.272	1.680	0.981	1.252	1.252
38	-0.094	0.181	-0.633	0.113	5.000	1.452	2.814	1.875	1.167	1.375	1.042	1.250	1.083
39	-0.041	-0.918	0.621	-0.484	6.000	0.633	1.043	2.787	0.213	1.827	0.993	1.127	1.073
40	-3.036	-1.027	0.144	-0.293	8.000	0.600	0.891	2.849	0.088	1.849	0.976	1.063	1.054
41	-2.879	0.349	0.432	-0.507	7.000	0.692	1.182	2.799	0.110	1.819	0.980	1.065	1.116
42	-0.915	-0.431	1.170	0.228	4.000	0.468	0.737	2.893	0.113	1.881	0.994	1.113	1.019
43	-2.915	-1.254	-0.067	0.491	6.000	0.271	0.351	2.927	0.044	1.946	0.995	1.020	1.005
44	2.210	-1.487	-0.761	-0.074	7.000	1.361	2.915	1.531	1.313	1.344	1.063	1.031	0.453
45	-0.497	0.547	-0.239	1.258	4.000	0.646	0.758	1.961	0.961	1.157	1.157	1.961	1.941
46	-0.908	1.164	-1.352	1.299	5.000	0.524	0.815	2.838	0.132	1.882	1.000	1.118	1.074
47	1.658	1.070	-1.772	-0.138	9.000	0.944	1.444	2.689	0.252	1.867	1.104	1.141	1.141
48	-2.831	-2.302	-0.326	0.940	5.000	1.049	2.370	2.485	0.182	1.515	0.833	1.045	1.121
49	0.365	-0.107	-0.685	0.751	6.000	0.693	1.122	2.780	0.228	1.961	1.173	1.205	1.142
50	0.824	1.708	-0.749	1.817	12.000	1.107	1.980	2.608	0.163	1.725	0.988	1.075	1.133
51	0.991	0.955	-1.167	1.852	7.000	1.487	3.401	2.840	0.220	1.380	0.700	1.160	1.160
52	1.628	0.789	0.470	-1.171	10.000	1.787	3.303	1.676	1.324	1.369	1.261	1.477	0.739
53	1.263	1.639	0.044	1.454	3.000	0.995	2.289	2.222	0.778	1.778	1.556	1.778	1.333
54	1.803	-0.315	-0.378	1.121	6.000	1.311	2.466	2.348	0.478	1.522	1.000	1.261	0.783
55	0.303	-2.452	-0.150	3.516	4.000	1.150	2.988	1.765	0.765	0.824	0.824	1.588	0.706
56	-1.994	0.069	-1.533	2.153	7.000	0.839	1.881	2.754	0.125	1.619	0.833	0.964	1.018
57	-2.848	0.215	0.467	-0.903	3.000	0.202	0.447	2.926	0.012	1.926	0.969	0.980	1.012
58	-2.651	-1.691	0.182	0.202	5.000	0.299	0.590	2.881	0.050	1.921	0.970	0.980	1.000
59	0.539	1.650	0.122	0.255	7.000	0.820	1.560	2.638	0.304	1.933	1.257	1.274	1.287
60	-0.315	-0.132	-0.513	0.037	6.000	0.752	1.284	2.726	0.282	1.752	1.017	1.248	1.231
61	0.611	1.915	0.245	0.004	5.000	0.612	0.978	2.773	0.100	1.827	0.991	1.091	1.082
62	-0.091	-1.117	-0.040	0.197	7.000	0.807	1.531	2.728	0.136	1.827	1.012	1.037	0.938
63	0.731	-1.843	1.357	0.506	10.000	1.567	3.230	2.388	0.866	1.806	1.493	1.388	1.328
64	1.384	0.468	0.848	0.366	5.000	0.414	0.708	2.899	0.055	1.881	0.963	1.028	1.046
65	1.597	-1.522	-0.626	-3.791	11.000	1.264	2.519	2.446	0.603	1.760	1.353	1.480	1.451

Mound ID	PC1	PC2	PC3	PC4	Species richness	Shannon diversity	Rao diversity	Colony size	Habitat pref.	Humidity requ.	Temp. requ.	Plasticity	Behaviour
66	-1.554	-0.595	1.850	-1.330	6.000	1.222	2.197	2.263	0.579	1.386	1.018	1.509	1.421
67	0.079	0.896	0.222	0.241	7.000	1.241	2.829	2.780	0.621	0.751	0.435	1.068	1.119
68	-1.466	0.677	1.300	-0.617	10.000	1.397	2.305	2.407	0.397	1.678	1.121	1.337	1.402
69	4.012	-2.897	2.454	0.439	6.000	0.968	2.048	2.139	0.778	1.028	0.889	1.667	1.639
70	-0.153	-2.292	1.937	1.077	7.000	1.608	3.414	2.429	0.381	1.143	0.714	1.143	1.190
71	1.188	1.232	0.106	-1.206	3.000	0.738	1.547	2.509	0.500	1.491	0.991	1.491	1.491
72	1.056	0.221	-1.195	-1.852	7.000	0.609	1.047	2.816	0.147	1.846	0.985	1.059	1.015
73	-2.544	-1.355	0.000	-0.630	7.000	1.020	2.120	2.099	0.863	1.490	0.977	1.038	1.057
74	0.726	-1.735	-2.710	-2.370	9.000	0.957	3.102	2.035	1.181	1.276	1.224	2.135	1.414
75	0.214	-1.310	-1.694	-2.562	5.000	0.532	0.823	2.851	0.135	1.950	1.092	1.128	1.078
76	-0.976	-0.791	-2.181	-0.939	1.000	0.000	0.000	3.000	0.000	2.000	1.000	1.000	1.000
77	-2.537	0.225	0.643	-0.584	10.000	1.205	2.754	2.403	0.149	1.507	0.791	0.866	1.015
78	-0.266	-0.266	2.127	0.015	6.000	0.973	1.886	2.597	0.176	1.630	0.924	1.101	1.134
79	0.751	1.778	-0.397	1.475	2.000	0.119	0.313	2.949	0.000	1.949	0.974	0.974	1.000
80	-1.697	-1.091	-1.040	0.745	2.000	0.693	2.690	1.500	0.500	1.000	0.500	1.500	1.000
81	-0.061	0.227	0.281	0.704	7.000	0.554	1.001	2.857	0.062	1.820	0.938	1.006	1.043
82	1.035	-0.735	1.302	0.885	4.000	0.230	0.286	2.933	0.045	1.955	1.000	1.022	1.022
83	1.013	0.020	-0.199	0.501	6.000	0.575	0.899	2.897	0.112	1.850	0.981	1.065	1.065
84	-0.892	0.284	-1.142	0.467	4.000	0.260	0.436	2.931	0.034	1.931	0.983	1.017	1.000
85	0.630	-2.098	-0.887	0.385	6.000	0.204	0.327	2.968	0.024	1.941	0.976	1.000	1.000
86	0.385	1.270	1.750	-1.266	6.000	1.247	3.029	2.804	0.601	1.059	0.627	1.176	1.072
87	1.148	-2.131	0.805	0.453	6.000	0.322	0.531	2.946	0.038	1.902	0.962	1.000	0.991
88	0.301	-0.062	0.017	0.621	6.000	1.240	2.043	2.200	0.673	1.755	1.445	1.627	1.682
89	-0.163	1.523	0.494	-0.338	7.000	1.175	2.191	2.360	0.618	1.303	0.933	1.528	1.461
90	-0.145	1.523	1.145	-1.128	6.000	0.682	1.230	2.768	0.192	1.755	0.974	1.166	1.172
91	-2.105	0.298	1.475	-2.146	2.000	0.693	0.779	2.000	1.000	1.500	1.500	2.000	2.000
92	-1.980	-1.988	0.304	0.768	6.000	0.481	0.899	2.817	0.033	1.850	0.933	0.950	1.000
93	-2.833	1.021	0.192	-0.918	7.000	0.502	0.759	2.898	0.091	1.920	0.977	1.057	0.989
94	-2.660	0.564	0.182	-0.169	3.000	0.113	0.175	2.975	0.019	1.975	0.997	1.016	0.981
95	1.084	0.937	0.120	-0.943	5.000	1.100	2.295	2.666	0.441	1.441	0.879	1.279	1.238
96	0.825	0.700	2.471	-0.720	3.000	1.004	2.535	2.077	0.000	1.346	0.808	0.808	1.000
97	1.329	1.570	-0.082	2.106	3.000	0.703	1.575	2.063	0.938	1.063	1.000	1.938	1.563
98	-2.037	0.978	-0.766	1.670	4.000	1.023	2.187	2.481	0.444	1.852	1.333	1.407	1.296
99	0.327	0.878	0.171	0.166	9.000	1.774	3.777	2.317	0.510	0.433	0.375	0.798	0.808

Mound ID	PC1	PC2	PC3	PC4	Species richness	Shannon diversity	Rao diversity	Colony size	Habitat pref.	Humidity requ.	Temp. requ.	Plasticity	Behaviour
100	3.658	-0.395	0.336	-0.468	5.000	1.205	1.567	1.200	0.650	0.950	0.950	1.100	0.900
101	0.768	0.957	1.347	-0.438	8.000	1.160	1.802	2.381	0.602	1.588	1.191	1.586	1.572
102	0.854	-1.830	-0.544	-0.681	5.000	1.157	2.314	2.319	0.043	1.511	0.851	0.957	1.021
103	0.249	-0.847	-0.464	0.297	4.000	0.559	1.084	2.893	0.107	1.786	0.929	1.036	1.036
104	1.311	-1.553	1.886	-0.068	2.000	0.271	0.516	2.077	1.000	0.923	0.923	1.923	1.923
105	1.327	1.942	2.321	-2.191	4.000	0.269	0.562	2.904	0.008	1.904	0.960	0.968	1.000
106	0.993	-0.900	-0.959	-1.452	6.000	0.435	0.570	2.901	0.094	1.922	1.021	1.089	1.078
107	4.124	-1.734	0.637	-0.769	2.000	0.287	0.729	3.000	0.083	1.833	0.917	1.000	1.000
108	-0.146	-2.747	-1.724	0.480	12.000	1.449	2.984	1.811	0.874	1.279	1.232	1.674	1.716
109	1.655	-0.400	-1.216	0.192	4.000	1.174	3.293	2.269	0.269	1.192	0.731	1.000	1.269
110	-1.335	1.418	0.433	0.099	5.000	0.735	1.794	2.642	0.063	1.665	0.875	0.938	1.040
111	-0.545	2.059	-0.456	0.267	3.000	0.865	2.850	2.423	0.115	1.538	0.885	0.885	1.115
112	-2.284	-0.695	0.362	0.710	10.000	1.092	2.391	2.670	0.144	1.557	0.856	0.938	0.866
113	-0.650	-1.475	0.637	-2.776	4.000	0.169	0.212	2.980	0.030	1.970	1.000	1.020	1.020
114	-1.443	1.707	0.128	-0.835	7.000	0.592	1.311	2.808	0.070	1.756	0.896	0.963	1.034
115	0.038	-2.024	-0.749	-0.872	6.000	0.821	2.146	1.796	1.083	1.907	1.750	1.759	1.648
116	-0.476	0.432	1.667	-0.490	5.000	0.807	1.233	2.690	0.197	1.845	1.000	1.113	1.000
117	1.199	0.695	0.122	-0.624	9.000	0.934	1.710	2.651	0.253	1.760	0.925	1.034	0.918
118	-0.765	0.690	0.450	-1.385	2.000	0.175	0.617	2.000	0.958	0.958	0.958	1.915	1.915
119	-0.170	1.100	-2.884	-2.473	5.000	1.561	6.097	1.500	1.500	2.000	1.667	1.833	1.000
120	0.888	1.289	-0.023	0.844	7.000	0.308	0.419	2.906	0.047	1.945	0.984	1.008	1.008
121	1.014	-0.586	0.645	1.504	5.000	0.766	1.571	2.376	0.616	1.354	0.981	1.597	1.601
122	1.113	-0.259	1.133	0.682	4.000	0.983	2.740	2.583	0.167	1.417	0.750	0.917	0.917
123	0.745	0.907	-0.846	-0.627	6.000	0.468	0.947	2.878	0.111	1.878	0.944	1.000	0.967
124	0.692	-1.754	-2.824	-1.258	7.000	1.778	5.301	2.615	1.692	1.308	1.154	1.385	1.385
125	0.464	0.714	-1.698	-0.200	6.000	0.496	1.396	2.116	0.027	0.259	0.134	0.150	0.134
126	0.467	1.929	-1.717	0.066	4.000	0.893	1.739	2.639	0.250	1.694	1.000	1.194	1.250
127	1.425	0.010	-1.421	-0.182	4.000	0.787	1.351	2.684	0.316	1.737	1.000	1.211	1.158
128	0.067	1.985	0.317	0.432	3.000	0.824	1.490	2.583	0.250	1.917	1.250	1.250	1.250
129	0.740	1.803	-0.880	2.271	4.000	1.197	3.926	2.071	0.214	1.214	0.786	0.857	1.071
130	1.516	0.854	-0.933	2.187	4.000	0.591	1.066	2.743	0.073	1.826	0.991	1.064	0.927
131	1.320	0.343	1.010	1.437	2.000	0.251	0.805	2.862	0.000	1.862	0.931	0.931	1.000
132	1.148	-1.130	0.769	0.655	6.000	0.757	1.260	2.776	0.204	1.776	0.980	1.122	1.143
133	0.892	1.368	2.196	-0.468	5.000	0.171	0.229	2.954	0.013	1.971	1.000	1.004	1.013

Mound ID	PC1	PC2	PC3	PC4	Species richness	Shannon diversity	Rao diversity	Colony size	Habitat pref.	Humidity requ.	Temp. requ.	Plasticity	Behaviour
134	0.678	1.592	1.063	0.187	6.000	1.405	2.383	2.127	0.302	1.508	1.079	1.286	1.317
135	-0.029	0.690	-3.074	0.027	4.000	0.711	1.304	2.720	0.280	1.800	1.000	1.120	0.960
136	0.728	-0.193	-0.815	-2.061	8.000	1.787	3.972	1.765	1.000	1.412	1.118	1.588	1.412
137	-0.107	0.251	-0.098	-0.761	4.000	0.298	0.515	2.870	0.022	1.935	0.989	0.989	0.978
138	0.760	1.453	-0.748	-1.118	5.000	1.026	2.238	2.121	0.848	1.152	0.909	1.636	1.576
139	-0.455	2.169	0.457	-0.885	3.000	0.659	1.192	2.160	0.760	1.200	1.000	1.760	1.760
140	0.290	2.287	-1.272	-0.714	4.000	0.936	1.736	2.367	0.100	1.700	1.067	1.100	1.100
141	0.291	2.262	-1.299	1.095	4.000	0.606	0.995	2.040	0.880	1.080	1.000	1.880	1.800
142	0.283	2.510	-0.546	0.870	3.000	0.645	1.276	2.545	0.030	1.758	1.000	1.030	1.030
143	0.244	2.469	0.601	-0.432	5.000	0.979	1.791	2.450	0.100	1.700	1.025	1.075	1.000
144	1.330	1.758	2.275	-0.943	6.000	0.196	0.287	2.951	0.033	1.967	0.997	1.015	1.006
145	-2.060	-0.592	-0.047	0.666	4.000	0.747	1.514	2.418	0.571	1.424	0.994	1.571	1.565
146	1.021	0.589	-1.670	-1.871	8.000	0.934	1.592	2.670	0.276	1.741	0.989	1.200	1.027
147	1.543	1.857	-0.213	0.836	7.000	0.446	0.768	2.848	0.110	1.924	0.995	1.043	0.957
148	1.640	0.647	3.043	-1.236	4.000	0.449	0.882	2.762	0.092	1.931	0.946	1.015	1.000
149	0.758	0.140	-1.185	0.754	6.000	1.551	2.577	2.239	0.500	1.609	1.217	1.500	1.152
150	0.797	0.848	1.275	-0.333	6.000	0.536	0.853	2.846	0.049	1.846	0.911	1.008	1.049
151	0.432	-0.727	-3.072	-1.295	10.000	1.987	4.090	1.867	1.311	1.778	1.467	1.556	0.978
152	-0.797	1.260	-0.038	-0.171	4.000	0.564	1.027	2.136	0.852	1.125	0.989	1.841	1.795
153	1.271	-0.720	-0.084	-2.538	4.000	0.348	0.595	2.880	0.133	1.907	0.987	1.013	0.987
154	0.814	0.215	0.525	-0.358	3.000	0.119	0.231	2.946	0.043	1.978	1.000	0.989	0.989
155	1.010	0.816	0.559	0.410	5.000	0.414	0.760	2.862	0.046	1.872	0.964	1.010	1.033
156	1.485	-0.199	0.996	0.099	2.000	0.683	1.467	2.429	0.571	1.429	1.000	1.571	1.571
157	0.745	1.071	0.592	0.034	5.000	0.210	0.300	2.936	0.032	1.962	0.994	1.013	1.013
158	0.071	0.126	2.387	-0.019	7.000	1.321	3.433	2.012	0.122	1.000	0.561	0.695	1.000
**mean**	0.057	0.032	0.000	-0.011	5.753	0.839	1.736	2.511	0.379	1.636	1.024	1.225	1.143
**SD**	1.462	1.2983	1.1945	1.1182	2.31772	0.4371812	1.1527	0.395601	0.3799	0.34133	0.248	0.31944	0.294518
**min**	-3.448	-4.018	-3.074	-3.791	1.000	0.000	0.000	1.200	0.000	0.259	0.134	0.150	0.134
**max**	4.124	2.510	3.043	3.516	12.000	1.987	6.097	3.000	1.692	2.161	1.965	2.166	2.000

Supplementary Table S5. Variance inflation factors calculated for the original predictors

**Original predictor**	**VIF**
Cover of herbaceous plants (%)	1.66
Cover of woody plants (%)	1.61
Mean litter thickness (cm)	1.60
Mean slope inclination (∘)	2.21
Mean vegetation height of herbaceous species (cm)	1.32
Mound area (m^2)	2.50
Mound height (m)	3.48
Percentage of croplands around the mounds (%)	6.44
Percentage of forests around the mounds (%)	2.94
Percentage of grasslands around the mounds (%)	2.72
Percentage of wetlands around the mounds (%)	3.51

Supplementary Figure S1 A. Association of the recorded 33 ant species with PC1 axis.

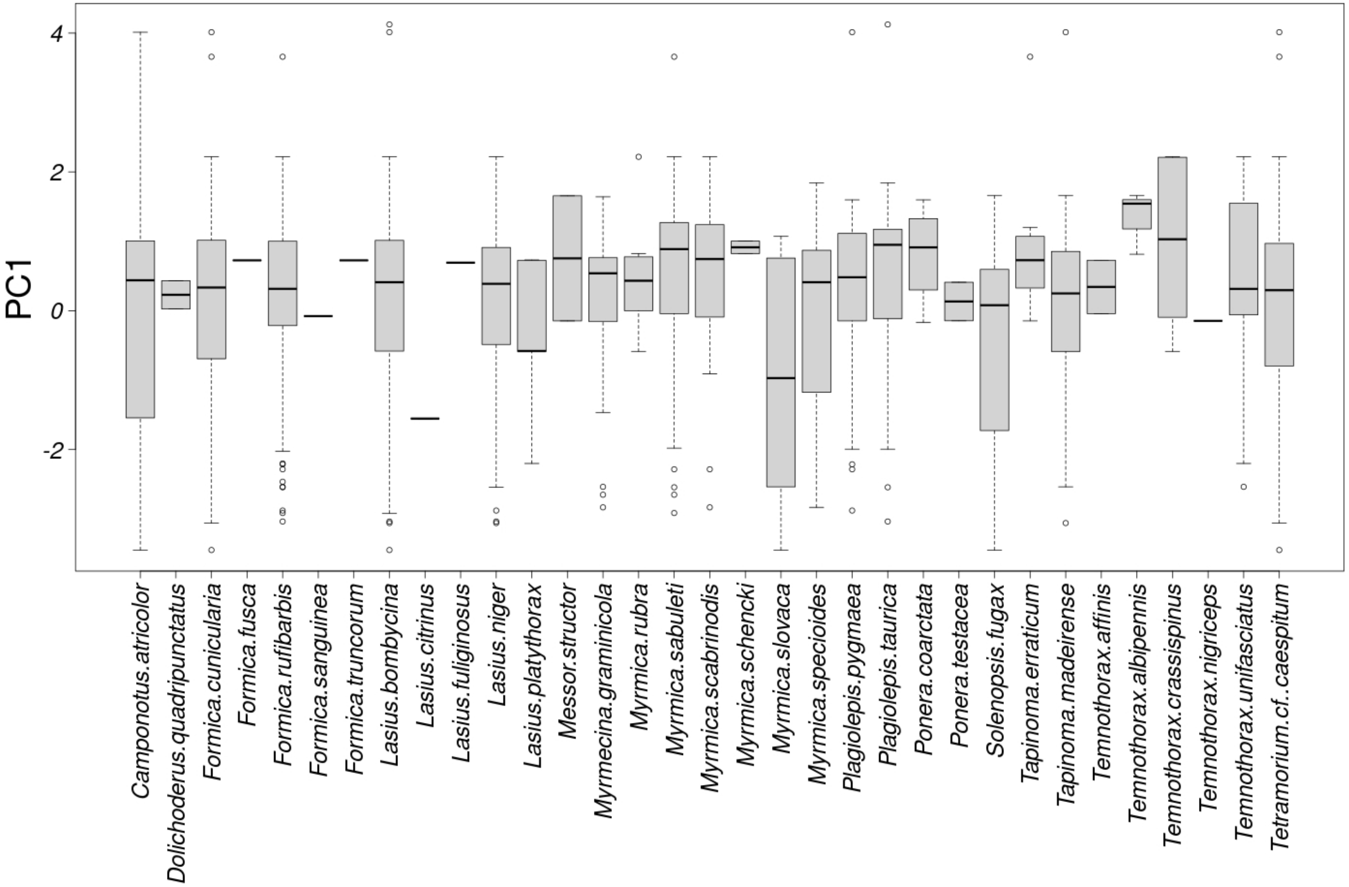

Supplementary Figure S1 B. Association of the recorded 33 ant species with PC2 axis.

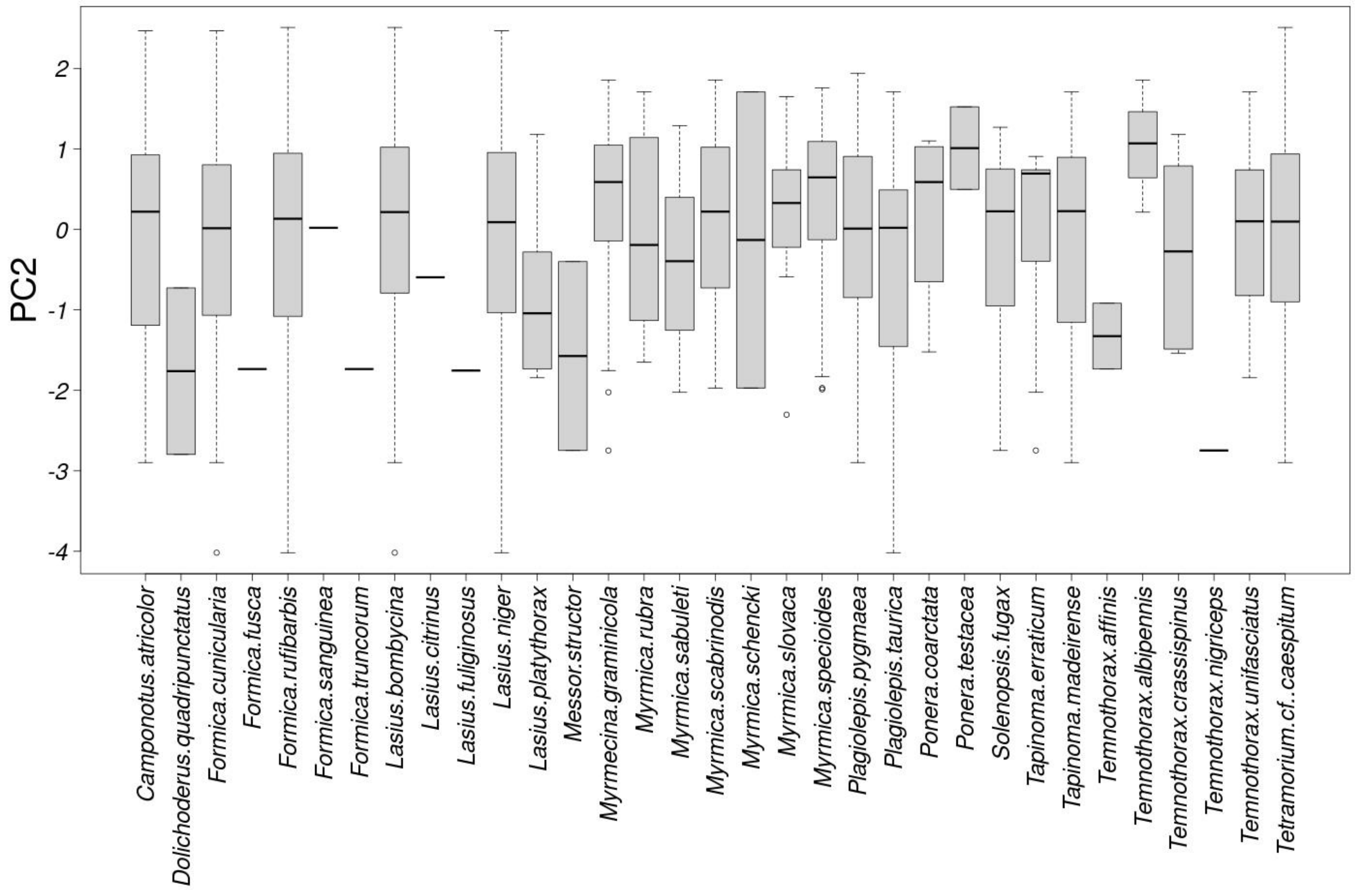

Supplementary Figure S1 C. Association of the recorded 33 ant species with PC3 axis.

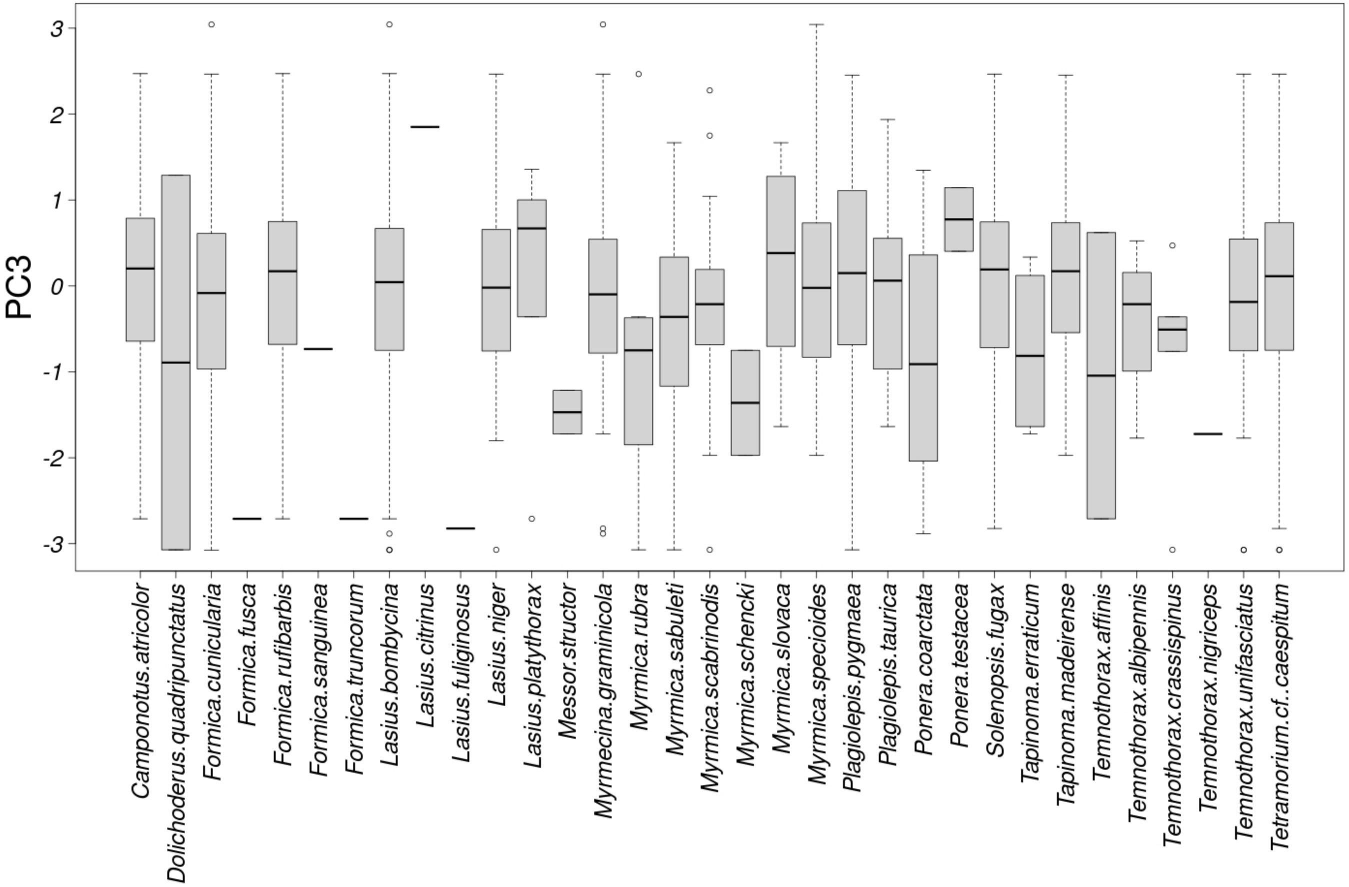

Supplementary Figure S1 D. Association of the recorded 33 ant species with PC4 axis.

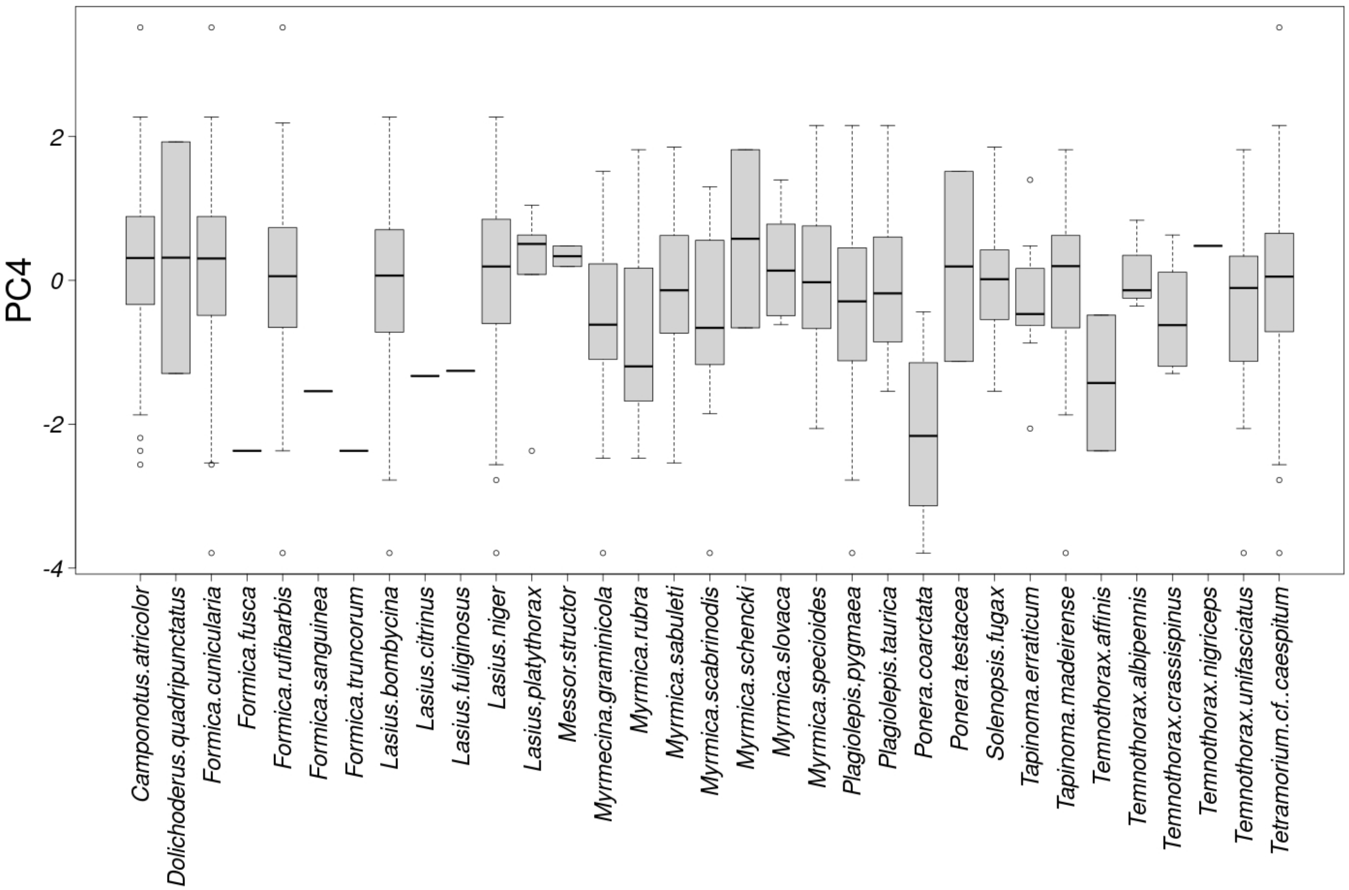

Supplement: Supplementary file 1 — Supplementary Information. [file 41598_2021_385_MOESM1_ESM.pdf]
